# Is Consumption Incurable?

**Published:** 1879-01-20

**Authors:** T. B. Greenley

**Affiliations:** Kentucky


### Southern Medical Record.

EDITORS:

T. S. POWELL, M.D. W. T. GOLDSMITH, M.D. R. C. WORD, M.D.

*R. C. WORD, M.D., Managing Editor.*

☞All Communications and Letters on Business connected with the RECORD must be addressed to the Managing Editor.

VOL. IX. ATLANTA, GA., JAN. 20, 1879. No. 1.

### ORIGINAL AND SELECTED ARTICLES.

### *"IS CONSUMPTION CONTAGIOUS?"*

BY T. B. GREENLEY, M. D., OF KENTUCKY.

This is a question being now frequently asked by many physicians, and should be considered by all as one of very great importance.

Dr. Holden, of Newark, N. J., contributed a very able article on this subject to the July number of the *American Journal of Medical Sciences*. He, conceiving it to be of very grave importance, took the pains to interrogate some five hundred physicians in the various States, about half of whom responded. Of these, one hundred and twenty-seven answered positively in the affirmative, and seventy-three in the negative, while twenty-one had not made any observations in that regard, and twenty-seven were not sure from what they had observed. Of the one hundred and twenty-seven who are convinced from their observations that consumption is contagious, many give detailed statements of cases having occurred in their immediate practice. Most of these are cases which seem to afford conclusive evidence wherein consumptive men married healthy women (whose family record afforded no scrofulous or tubercular taint), and vice versa.

Some very striking instances are related: thus, Dr. Hoff, of Pomroy, Ohio, says: "Recently I knew a wife to take and die of consumption; in one year it was fully developed in her husband. He married, however, and died within a year thereafter. His second wife followed him with the same disease, and also their babe."

Dr. L. McDonald, of Flemingsburg, Ky., says "he knew a man who had all the external appearance of a tuberculous diathesis, who married a healthy girl of eighteen. In three years (after the birth of her second child), she died of tuberculosis. He married in a year and a half again, and to an exceedingly robust woman of a family without taint. In less than two years, she died of unmistakable pulmonary consumption. The third time he married a healthy woman. He himself died in twelve months after his third marriage, with consumption, and his widow followed him in six months with the same disease."

Several other reports equally striking might be copied, but these two will be sufficient to indicate the character of others.

I can report only two cases wherein I was convinced that contagion or *infection* played a conspicuous part.

M. G., whose family record was without taint, married a woman of tuberculous diathesis, who died of consumption, and in a few years her husband had the disease developed, and finally died. He was a very devoted husband, and nursed his wife faithfully.

Mr. S., who was of a tuberculous family, married a woman of good family record. He was sick some two or three years with consumption before he died, his wife being his principal nurse. She soon succumbed to the same disease.

I could give several other cases of similar character, in the practice of some of my neighbors, and doubt not but one or more cases might be reported by a majority of physicians who have been practicing so long as ten years.

A great many physicians do not believe in the doctrine that tuberculosis of the lungs is contagious; and I presume there are but few, if any, who believe that it is as much so as some of the well-known contagious diseases. Small-pox is put down as the most contagious of all diseases, and yet a few persons escape taking it, although frequently exposed to it. We must admit that many cases of consumption die without the disease being communicated to others, but when so many cases come under the observation of medical men, wherein to all appearances we cannot account for the disease except through contagious influences, we as honest men surrender our preconceived notions.

I formerly was of the opinion that the only way consumption was contagious was from the husband to the wife through impregnation—her blood becoming contaminated through the placenta. Of course everybody believes in the transmissibility of the disease from parent to child. Yet this is not a universal law, as we not unfrequently see offspring of one consumptive parent entirely escape the disease. There is no disease that is universally contagious—small-pox coming the nearest; and because we frequently see cases of consumption nursed and treated for years without it *being* communicated to the attendants, we cannot say positively that it is not contagious. There are many persons who never take measles, whooping-cough, mumps, etc., although frequently exposed, yet we must not say these diseases are not contagious.

If we admit that consumption is contagious, the question then arises, How is it so? Is it communicated by contact of person, or through the respiratory organs, by breathing the contaminated air that has been breathed and rebreathed by the patient? Dr. Holden believes that the disease-poison is absorbed by the skin, by sleeping with the patient. He thinks the deleterious principle is contained in the exhalations from the surface of the sick, in those colliquative sweats to which consumptives are subject, and ignores the factor of foul air. His argument is plausible and ingenious. He cites the history of the great Brompton Hospital for consumptives, for the last twenty years, which shows only two cases of the disease developed in that institution, and remarks that we may dismiss the idea of danger when perfect ventilation is observed. He also believes that the disease may be communicated by wearing the clothing of the sick. Of course we have no evidence furnished us by the Brompton Hospital record, except of a negative character. The nurses did not sleep in the same beds with the patients, and as it is presumed the wards were well ventilated, we could not expect the disease to be communicated either by respiring foul air, or by contact in sleeping. It occurs to me that of the two theories of the manner of communication, that of breathing rebreathed air is the most reasonable. It is almost the constant custom with consumptives, after becoming confined to the house, to keep their rooms close—they are afraid of *fresh air*, and neglect ventilation, one of the most important elements of treatment. Of course the nurse is subject to the deleterious influences of a close and contaminated atmosphere; and if the case should be very protracted (which is very common), the general health of the attendant becomes more or less vitiated and impaired, and thereby more readily succumbs to the contagious influence; and I cannot but believe this is effected through the lungs. In fact, I believe *all contagious* as well as malarial diseases (except those by contact of diseased parts), are communicated by respiring contaminated air.

The question may be asked, What is the character of this contagious element that may produce consumption? It is doubtless morbific matter exhaled, no doubt, both from the lungs and skin of the patient, but as to its chemical or physiological characteristics it would be hard to determine. It is perhaps of a microphitic nature, like the elements of other contagious diseases, and which cannot be made tangible to the senses. This particular matter, no doubt, is the product of metamorphosed tissue taking place in the lungs, and from its results must be tubercular matter in infinitesimal portions.

It is argued by anti-contagionists that we can account for the disease affecting nurses, etc., who attend on consumptives, by loss of rest, distress of mind, close confinement, etc., but if these were the only elements in action, why should not some other disease be the result?

There must be some specific influence exerted where so many cases occur; they cannot be the result of mere accident. The law let down by the Great Architect in the earliest dawn of creation, that like should beget like, hardly ever varies, and where it does, it is only an exception to the law.

My object in writing this article was not so much to discuss the particular manner in which consumption may be communicated from the sick to the well, as to draw the attention of the profession to the fact of its contagious character; and, if possible, to elicit some suggestions in the way of prophylaxis.

Hygiene and state medicine have done a great deal in the prevention of some diseases, and I think much might be done to curtail the mortality resulting from tuberculosis.

We have, within the past century, by the use of preventive means, lengthened human life five years in a generation, and this has been effected by the adoption of measures purely hygienic, and mainly independent of legislative enactments. This has consisted in ventilation, drainage, sewerage, etc., and in the arrest of the spread of epidemics. If such gratifying results have been obtained so easily, how much might be accomplished by legislative action?

Now we have seen that in a period of over twenty years in the great consumption hospital of Brompton, only two cases of tuberculosis were developed in the institution. We must attribute this extraordinary exemption to the admirable system of ventilation observed. It is to be hoped that when such beneficial results ensue from such simple means, that attention will be given this very important matter. As before remarked, it is a very common, yea, almost a universal practice with persons affected with consumption, to keep their rooms closed, fearing the least breath of air, as they say, will give them cold. Now it seems to me that a little tuition put into practice on the part of the attending physician, would soon convince the patient that not only no danger would ensue from proper ventilation, but on the other hand it would be attended with great utility, not only to himself, but to his attendants. We find a great many consumptives in very badly constructed houses, and ordinarily not well adapted to ventilation, but the genius of the medical attendant should not be heavily taxed to devise means to furnish fresh air to the invalid's room. We should observe this sanitary measure, not so much with the expectation of restoring the health of the patient, but to prevent deleterious consequences to the attendants. I make these remarks on the premises that but few consumptives reocver after once taking to their rooms.

But might not a great deal be done in the way of sanitation, by proper ventilation, combined with other hygienic measures, in the prevention of the developement of the disease in those hereditarily predisposed? This is a question of momentous import, when we consider the vast mortality resulting from consumption in this country. In the report of the State Board of Health of Massachusetts for the year 1877, we notice that this disease stands first as the cause of death in each decade from the age of fifteen to seventy, and caused more than one sixth of the entire mortality of the State—about seventeen per cent. It may be that owing to the great number of persons engaged in the manufacturing establishments of that State, this disease is more prevalent than in some others of the Northern States. In Michigan, the mortality of consumption ranges from eleven to fourteen per cent. of the entire deaths.

Now cannot something be done in the way of preventive means to arrest or at least modify the progress of this terrible destroyer of human life? We think the profession have it to some extent in their power to do something in this way, independent of legislative means. It would be considered very arbitrary to speak of legislative enactments to prevent the inter-marriage of tubercular subjects, and thereby strike at the root of this great enemy to the human race; but doubtless this is the only means by which it could be to any great extent eradicated from our midst.

